# Spontaneous Bilateral Renal Forniceal Rupture Secondary to Acute Urinary Retention in a Patient with Prior Prostate Radiotherapy: A Case Report

**DOI:** 10.3390/reports9020184

**Published:** 2026-06-12

**Authors:** Timoleon Giannakas, Dimitrios Deligiannis, Panagiotis Mitsos, Anna Papakonstantinou, Marios Stavropoulos, Aris Kaltsas

**Affiliations:** 1 Third Department of Urology, Attikon University Hospital, School of Medicine, National and Kapodistrian University of Athens, 12462 Athens, Greece; tgiannakas@gmail.com (T.G.); stamarios@yahoo.gr (M.S.); 2 Department of Urology, General Children’s Hospital “Agia Sofia”, Thivon 1 and Papadiamantopoulou, 11527 Athens, Greece; mitsospanagiotis@yahoo.com (P.M.); annapapak86@gmail.com (A.P.)

**Keywords:** renal forniceal rupture, urinary retention, bladder outlet obstruction, urinoma, CT urography, prostate radiotherapy, post-obstructive diuresis, percutaneous nephrostomy

## Abstract

**Background and Clinical Significance:** Spontaneous renal forniceal rupture is an uncommon complication of obstructive uropathy and is classically associated with ureteric calculi rather than distal urinary retention. Bilateral retention-related rupture appears to be exceptionally rare and may be diagnostically challenging when renal function begins to improve after bladder decompression; **Case Presentation:** An 82-year-old man with a history of prostate cancer treated five years earlier with external beam radiotherapy and androgen deprivation therapy presented with acute abdominal pain radiating to both flanks and inability to void. Bedside ultrasonography showed urinary retention and bilateral hydronephrosis, and a 16-Fr Foley catheter drained 900 mL of urine. Admission evaluation showed severe acute kidney injury, microscopic hematuria, minimal leukocyturia, and elevated inflammatory markers. Post-obstructive diuresis developed after bladder decompression. CT urography with excretory-phase imaging on hospital day 3 demonstrated severe bilateral hydroureteronephrosis with bilateral renal forniceal rupture and associated urinomas, including a larger left-sided collection extending toward the psoas compartment. Bilateral percutaneous nephrostomies were placed on hospital day 4 for upper-tract diversion. Immediate nephrostography showed no active contrast extravasation. At one-month follow-up, combined CT and nephrostographic assessment confirmed complete resolution of the bilateral urinomas without persistent leak, and the nephrostomy tubes were removed; **Conclusions:** This case suggests that urinary retention in an older man with prior prostate radiotherapy may reflect radiation-associated outlet pathology and/or impaired detrusor function rather than simple prostate enlargement. Delayed-phase CT urography was essential for diagnosis, and active bilateral diversion was justified by bilateral rupture, acute kidney injury, and the extent of urinary extravasation. The report expands the limited PubMed-indexed literature on retention-related upper urinary tract rupture and supports cautious follow-up aimed at defining the underlying mechanism of retention.

## 1. Introduction and Clinical Significance

Spontaneous disruption of the upper urinary tract is an uncommon but clinically important event that usually reflects obstruction with rapid transmission of pressure to the collecting system [1,2,3,4,5,6]. In pressure-related cases, the most vulnerable site is the calyceal fornix, where increased intrapelvic pressure may exceed local tissue tolerance and permit urine to escape into the peripelvic or perirenal space [1,2,3,4,5]. The initial extravasation can behave as a decompressive “pop-off” mechanism, but it can also lead to symptomatic urinoma, persistent leakage, infection, ongoing pain, and renal dysfunction if the underlying obstruction is not recognized and relieved [3,5,6]. Because symptoms overlap with far more common causes of flank or abdominal pain, the diagnosis may be delayed unless clinicians maintain a specific suspicion for upper urinary tract leakage in the setting of hydronephrosis or obstructive nephropathy [5,6].

Distal ureteric calculi remain the predominant cause of renal forniceal rupture, while non-stone etiologies are distinctly uncommon [1,2,3,4,7,8,9,10,11]. In the largest CT-based retrospective series identified in PubMed, ureteric stones accounted for 74.1% of cases, whereas bladder outlet obstruction accounted for only 0.9% [4]. This distribution is important because acute urinary retention is common in older men, yet rupture of the renal fornix or other upper urinary tract structures remains exceptionally rare in that context [4,12,13,14,15,16,17,18]. When upper tract rupture does occur after retention, the event raises immediate questions regarding the severity and chronicity of distal obstruction, the possibility of high-pressure retention physiology, and the need to exclude more common alternative explanations such as ureteric calculi, malignant compression, retroperitoneal fibrosis, or iatrogenic injury [1,2,3,4,5,6,9,10,11].

The present case is especially instructive because the patient had undergone external beam radiotherapy and androgen deprivation therapy for prostate cancer five years earlier, but his prostate volume at presentation was only approximately 30 mL. In this setting, attributing retention solely to benign gland enlargement is not persuasive. Radiation-associated urethral stricture disease, bladder neck contracture, reduced outlet compliance, chronic ischemic fibrosis, and secondary detrusor dysfunction are all biologically plausible late explanations for urinary retention and upper tract deterioration after prostate cancer treatment [19,20,21,22,23,24]. Importantly, urinary adverse events after pelvic radiotherapy may present years after treatment and may range from irritative lower urinary tract symptoms to recurrent infection, hematuria, outlet obstruction, fistula formation, urethral stricture disease, bladder-neck stenosis, or frank retention [19,20,21,22,23,24].

This case report describes an 82-year-old man who developed acute kidney injury, bilateral hydroureteronephrosis, and spontaneous bilateral renal forniceal rupture with associated urinomas after acute urinary retention. The case is presented in accordance with the CARE guideline [25]. Emphasis is placed on the likely mechanism of urinary retention, the chronology of decompression and imaging, the rationale for bilateral upper tract diversion, and the limited PubMed-indexed literature on retention- or bladder outlet obstruction-related upper urinary tract rupture.

Clinical Significance: This case highlights that abdominal or flank pain and acute kidney injury in a patient with urinary retention should prompt consideration of upper urinary tract rupture even when lower urinary tract decompression has already been achieved. It also emphasizes that a relatively small prostate does not exclude clinically significant outlet dysfunction in a previously irradiated patient, and that delayed-phase CT urography is central to defining the site, laterality, and extent of urinary extravasation before selecting conservative management or active diversion [1,2,3,4,5,6,7,8,26,27].

## 2. Case Presentation

### 2.1. Patient Information and Emergency Assessment

An 82-year-old man with a history of prostate cancer presented to the emergency department of a tertiary referral hospital with acute-onset lower abdominal pain radiating bilaterally to the flanks and an inability to void. The pain had increased progressively over several hours and was described as constant rather than colicky. He also reported marked suprapubic fullness and discomfort during repeated unsuccessful attempts to urinate. He denied fever, gross hematuria, nausea, vomiting, bowel obstruction symptoms, recent trauma, or recent instrumentation, but described a several-month history of nocturia, progressively weaker stream, intermittency, and incomplete bladder emptying that had intensified during the week before admission.

On arrival, the patient was mildly confused but cooperative. His vital signs were stable: blood pressure 145/75 mmHg, pulse 89 beats/min, respiratory rate 21 breaths/min, oxygen saturation 96% on room air, and temperature 36.3 °C. Abdominal examination demonstrated suprapubic distension with diffuse lower abdominal tenderness and bilateral flank discomfort without rebound tenderness or generalized peritonism. Digital rectal examination showed a non-nodular prostate of modest size without marked tenderness.

His relevant history included prostate adenocarcinoma diagnosed five years earlier and treated with external beam radiotherapy and androgen deprivation therapy. Previous pathology had shown Gleason score 7 (4 + 3), corresponding to ISUP grade group 3. No prior upper urinary tract rupture, nephrolithiasis, or major reconstructive urological procedure was documented. At presentation, the working differential diagnosis included acute urinary retention with obstructive nephropathy, complicated urinary tract infection, ureteric obstruction from calculus or malignancy, and less likely an intra-abdominal or vascular emergency masquerading as urological pain.

### 2.2. Initial Laboratory and Bedside Evaluation

Initial urinalysis showed microscopic hematuria and minimal leukocyturia, with 23 red blood cells/high-power field and 3 white blood cells/high-power field. This degree of leukocyturia was minimal and was not considered diagnostic of clinically significant pyuria. The white blood cell count was 9.5 × 10^9^/L, and C-reactive protein was 117 mg/L. Serum creatinine on admission was 5.2 mg/dL, indicating severe acute kidney injury. Electrolytes did not show an immediately life-threatening abnormality, but the combination of renal dysfunction, urinary retention, and bilateral flank pain raised concern for substantial obstructive uropathy. The most recent outpatient creatinine available before admission was 1.1 mg/dL, indicating that the renal impairment was acute rather than chronic.

Bedside ultrasonography demonstrated a distended urinary bladder with an estimated volume of approximately 700 mL and bilateral hydronephrosis. Prostate volume was estimated at 30 mL. A 16-Fr Foley catheter was inserted without technical difficulty and drained 900 mL of urine. The ease of catheterization made a dense fixed anterior urethral stricture less likely at the bedside, although it did not exclude a short or compliant post-radiation narrowing at the bladder neck or prostatic urethra. At this stage, the leading diagnosis was acute urinary retention complicated by bilateral obstructive uropathy and acute kidney injury.

### 2.3. Early Inpatient Course After Bladder Decompression

The patient was admitted for close monitoring of urine output, renal function, fluid balance, and decompression-related complications. During the first 24 h after bladder drainage, he developed post-obstructive diuresis. Peak urine output was approximately 3.7 L in the first 24 h and was managed with isotonic intravenous fluid replacement titrated to urine output, daily weight assessment, and serial electrolyte measurements. This evolution was clinically important because the combination of a substantial retained volume and markedly elevated creatinine placed him at increased risk for significant diuresis after decompression [26,27].

Serum creatinine improved progressively after catheterization; however, the clinical course remained concerning. The creatinine trend was 5.2 mg/dL on admission, 3.4 mg/dL on day 1, 2.1 mg/dL on day 2, 1.5 mg/dL on day 3, and 1.1 mg/dL by day 4. Despite this biochemical recovery, persistent bilateral flank pain and the severity of sonographic hydronephrosis raised concern that lower urinary tract decompression alone might not fully explain or resolve the upper tract process. The team therefore escalated the diagnostic work-up to CT urography with delayed excretory-phase imaging.

Because markedly elevated inflammatory markers were present at admission, empiric intravenous antibiotics were initiated while microbiological studies were pending. Urine and blood cultures remained negative, and empiric ceftriaxone was discontinued after 5 days once the patient remained afebrile, leukocyte counts stayed normal, and inflammatory markers declined. The inflammatory response was therefore interpreted as reflecting urinary stasis and/or a local inflammatory reaction associated with urinary extravasation rather than overt urosepsis.

### 2.4. Cross-Sectional Imaging and Diagnostic Interpretation

CT urography with delayed excretory-phase imaging was performed on hospital day 3, after Foley catheter placement and partial biochemical recovery, because of persistent pain, bilateral hydronephrosis, and concern for a superimposed upper tract complication. The scan demonstrated severe bilateral hydroureteronephrosis and bilateral urinary extravasation arising from the renal fornices, consistent with spontaneous bilateral renal forniceal rupture (Figure 1). Both ureters appeared to be markedly tortuous. Because the rupture was demonstrated after catheterization, the exact bladder configuration at the moment of forniceal failure could not be reconstructed. At CTU, the Foley catheter remained in situ and the bladder was decompressed/underdistended, with apparent circumferential wall thickening/trabeculation and no CT evidence of bladder wall rupture, vesical contrast extravasation, or an intravesical obstructing mass/clot (Figure 1B). On the left, contrast leak from the collecting system was associated with a sizeable urinoma extending inferiorly along the anterior pararenal space toward the psoas compartment. On the right, there was a smaller perirenal urinoma associated with a second forniceal leak.

The overall imaging pattern favored a pressure-transmission injury from distal outflow obstruction rather than a primary focal upper tract lesion. The left-sided collection measured approximately 15 cm × 6 cm on axial imaging with caudal extension along the left psoas margin, whereas the right perirenal collection measured approximately 2.6 cm × 4.7 cm. No ureteric calculus, pelvi-ureteric junction obstruction, distal ureteric mass, retroperitoneal fibrosis, or bulky pelvic lymphadenopathy was identified. There was also no radiologic evidence of locally recurrent or metastatic prostate cancer causing distal ureteric compression, and the most recent oncological follow-up had not suggested biochemical recurrence. These findings materially strengthened the causal link between urinary retention, bilateral hydroureteronephrosis, and the bilateral upper urinary tract rupture.

In retrospect, the presentation likely represented acute retention superimposed on evolving lower urinary tract dysfunction. The abrupt inability to void was one of the main symptoms that brought the patient to hospital, but the bilateral hydronephrosis, severe renal impairment, and post-obstructive diuresis suggest that raised lower urinary tract pressure had already been affecting the upper tracts before admission. This interpretation became important when deciding whether continued Foley drainage alone would be sufficient or whether additional upper tract diversion should be undertaken. Accordingly, although bilateral rupture was diagnosed only on day 3 after bladder decompression, the available findings favor rupture that had already occurred before hospital imaging and probably before or around the initial decompressive period during high-pressure retention, rather than a new injury caused by Foley catheter drainage.

### 2.5. Therapeutic Intervention

Management options considered after CTU included ongoing bladder drainage with observation, retrograde ureteral stenting, antegrade stenting, percutaneous nephrostomy, or combined drainage of the collecting system and any persistent urinoma. Conservative treatment was considered potentially inadequate because the case was complicated by bilateral rupture, marked hydronephrosis, severe acute kidney injury at presentation, an elevated inflammatory response, and a relatively large left-sided urinoma. Although some uncomplicated cases of forniceal rupture can be managed conservatively [3,7,8], this patient was judged to have a sufficiently high-risk profile to justify active bilateral upper tract decompression.

On hospital day 4, bilateral percutaneous nephrostomy tubes were inserted by interventional radiology team without immediate complication. Two 8.5-Fr pigtail nephrostomy catheters were placed. Immediate bilateral nephrostography demonstrated opacification of both pelvicalyceal systems without ongoing active contrast extravasation (Figure 2 and Figure 3). This absence of leak on nephrostography was interpreted as pressure-dependent cessation or rapid sealing after decompression and as a consequence of the different timing and contrast-injection conditions compared with the prior delayed CTU, rather than as a contradiction of the CTU diagnosis. Because the collecting systems decompressed satisfactorily and no persistent leak was visualized, antegrade ureteral stents were not inserted at that setting.

Retrograde stenting was discussed but deferred, as the percutaneous nephrostomy approach provided rapid and symmetrical bilateral decompression and avoided immediate manipulation of the tortuous ureters, which could have interfered with prompt stent placement. Separate percutaneous drainage of the left urinoma was also not performed because the collection was expected to regress after proximal diversion and showed no radiological features suggestive of abscess formation.

### 2.6. Clinical Outcome and Follow-Up

After upper tract diversion, the patient remained hemodynamically stable and afebrile. Flank pain improved progressively, urine output normalized, and inflammatory indices continued to fall. He was discharged on hospital day 7 with the Foley catheter and both nephrostomy tubes left in situ, together with a short oral antibiotic tail and instructions for close outpatient follow-up. Renal function had normalized by the time of discharge.

At one-month follow-up, cross-sectional imaging combined with bilateral nephrostogram demonstrated complete resolution of the urinomas and no persistent urinary extravasation. Both nephrostomy tubes were therefore removed. Serum creatinine remained 1.1 mg/dL at follow-up.

Because the precise mechanism of retention could not be fully resolved during the acute admission, lower urinary tract reassessment was arranged after the upper tract problem had settled. Flexible cystoscopy performed after recovery showed no fixed anterior urethral stricture, mild fibrotic narrowing at the bladder neck/prostatic urethra consistent with post-radiation change, and a trabeculated bladder without visible tumor. A later trial without catheter was successful, with a post-void residual of approximately 120 mL; the patient was maintained on alpha-blocker therapy and scheduled for further functional assessment to clarify the contribution of detrusor underactivity.

A concise day-by-day summary of the clinical course is shown in Table 1. This timeline was included to improve chronological clarity and to align the report more closely with CARE-style reporting expectations [25].

## 3. Discussion

### 3.1. Mechanism of Rupture in the Setting of Urinary Retention

This case illustrates a rare endpoint of distal urinary outflow failure: bilateral renal forniceal rupture with bilateral urinomas after acute urinary retention. The pathophysiology is clinically coherent. When intravesical pressure remains sufficiently high and that pressure is transmitted through dilated ureters to the upper tracts, intrapelvic pressure may exceed the threshold tolerated by the calyceal fornix, allowing urine to dissect into the peripelvic or perirenal space [1,2,3,4,5,6]. In many patients, the leak behaves as a decompressive “pop-off” phenomenon, but this should not obscure the fact that renal function may already be threatened by the underlying obstruction or by secondary effects of the resulting urinoma, including pain, infection, or pressure-related distortion of renal anatomy [3,5,6].

Most descriptions of spontaneous forniceal rupture focus on ureteric calculi, in which a relatively abrupt rise in pressure develops above a focal obstructive point [1,2,3,4,7,8]. Retention-related rupture is less intuitive because the obstructive lesion is distal and often evolves over a longer interval. However, descriptions of obstructive uropathy and retention-related upper tract rupture show that persistently elevated bladder storage pressure can produce bilateral hydroureteronephrosis, progressive renal impairment, and striking post-decompression diuresis [17,18,26,27]. The present patient likely occupied an intermediate pathophysiologic position between acute retention and high-pressure chronic retention: sudden painful inability to void prompted presentation, yet the bilateral hydronephrosis, marked creatinine elevation, and subsequent post-obstructive diuresis strongly suggest that elevated lower urinary tract pressure had already been affecting the upper tracts before hospital presentation [18,26,27].

This distinction matters because it helps explain why bilateral rupture could occur even in the absence of ureteric calculus. Acute-on-chronic escalation in outlet resistance may have converted compensated upper tract dilation into frank forniceal failure on both sides. The larger left urinoma may simply reflect asymmetry in local compliance, forniceal vulnerability, or the exact timing of pressure release rather than a different distal cause. From a clinical standpoint, the bilateral nature of the rupture supports a global pressure-transmission mechanism rather than a primary unilateral ureteric event.

### 3.2. Likely Cause of Urinary Retention in This Patient

The proximate cause of rupture was most likely urinary retention, but the more nuanced issue concerns the cause of retention itself. Simple benign prostatic enlargement is not the most convincing explanation because the prostate volume was only approximately 30 mL and catheterization was achieved without marked difficulty. Contemporary male LUTS/BPH guidance emphasizes that symptoms and obstruction may be multifactorial and that prostate size alone does not fully define functional outlet obstruction; accordingly, a modest prostate volume weakens the argument for simple volume-driven obstruction as the sole mechanism in the present case [28,29,30]. In contrast, prior prostate radiotherapy introduces several biologically plausible explanations for outlet dysfunction, including bladder neck fibrosis, prostatic urethral narrowing, membranous urethral stricture, chronic ischemic tissue change, reduced outlet compliance, and secondary detrusor dysfunction [19,20,21,22,23,24]. These late complications may emerge years after treatment and may present with worsening lower urinary tract symptoms, incomplete emptying, recurrent infection, hematuria, or frank retention [19,20,21,22,23,24]. Recurrent malignant disease must also remain in the differential diagnosis; therefore, biomarkers, imaging findings, and longitudinal oncological follow-up should be interpreted together rather than in isolation.

The follow-up cystoscopic findings in this patient—mild bladder neck/prostatic urethral narrowing with post-radiation fibrosis but no dense anterior urethral stricture or tumor—fit well within this framework. Even if impaired detrusor contractility also contributed, the central message remains unchanged: in older men with prior prostate radiotherapy, urinary retention should not be attributed automatically to gland size alone. The outlet may be functionally or anatomically compromised despite a relatively small prostate, and such patients may develop upper tract consequences out of proportion to what prostate size alone would predict.

Equally important is the exclusion of major alternative explanations. CT urography did not show ureteric calculi, pelvic or retroperitoneal mass effect, or other focal upper tract obstruction, and follow-up evaluation did not suggest recurrent urothelial or prostate malignancy. No history of recent instrumentation or trauma existed. This process of exclusion materially strengthens the claim that retention with distal outlet pathology was the operative mechanism. Nevertheless, the manuscript should remain appropriately cautious and acknowledge that the relative contributions of post-radiation outlet narrowing, and detrusor underactivity cannot be quantified definitively without comprehensive functional testing.

### 3.3. Imaging and Diagnostic Strategy

This case underscores the pivotal diagnostic role of contrast-enhanced CT with delayed or excretory-phase imaging in suspected urinary extravasation [1,2,3,4,5,6]. Bedside ultrasonography was useful for confirming urinary retention and hydronephrosis, as well as excluding significant bladder pathology; however, it was unable to assess the integrity of the collecting systems or determine the extent of urinary extravasation. Delayed-phase CT urography demonstrated both the site and laterality of extravasation, identified the associated urinomas, and excluded common competing causes such as ureteric stones or malignant compression. In practical terms, CTU transformed the problem from generic obstructive acute kidney injury to a clearly defined bilateral upper urinary tract leak requiring management revision [5,6,27].

The imaging findings were especially helpful because symptoms after bladder decompression can be misleading. Had the improving creatinine level been interpreted in isolation, clinicians might have concluded that Foley drainage alone had resolved the obstructive event. Instead, persistent flank pain prompted further imaging, revealing a more complex underlying process. This highlights an important clinical point: biochemical improvement following catheterization does not exclude ongoing upper urinary tract complications, particularly in the presence of persistent pain, fever, elevated inflammatory markers, or disproportionate hydronephrosis.

### 3.4. Rationale for Bilateral Percutaneous Nephrostomy

Management of spontaneous forniceal rupture is individualized because the evidence base consists primarily of observational studies, literature reviews, and case reports rather than randomized comparisons [3,7,8]. In uncomplicated cases—particularly small leaks with limited urinoma, controlled pain, stable renal function, and no infection—conservative treatment with bladder drainage, analgesia, antibiotics when indicated, and observation may be sufficient [3,7,8]. By contrast, intervention is favored when there is uncontrolled pain, sepsis, solitary kidney, substantial renal impairment, persistent obstruction, or sizable urinoma [3,7,8].

Several features argued against simple observation. First, the rupture was bilateral. Second, presentation included severe acute kidney injury. Third, the left-sided urinoma was relatively large and extended toward the psoas compartment. Fourth, a clear inflammatory response was present at admission, even if overt sepsis was absent. Taken together, these features placed the patient at higher risk of prolonged leakage, secondary infection, and delayed recovery if upper tract pressure was not reduced promptly. Bilateral percutaneous nephrostomy therefore offered rapid, direct, and symmetrical decompression.

Retrograde ureteral stenting would also have been a reasonable option in certain settings; however, percutaneous nephrostomy (PCN) offered specific advantages in this case. It avoided urgent retrograde manipulation through a potentially irradiated outlet, as well as catheterization of two tortuous ureters, provided direct access to both collecting systems, and enabled immediate nephrostographic confirmation of decompression. The fact that no separate drain was required for the left urinoma also fits established practice, since not every urinoma requires direct drainage if the source pressure is controlled and the collection is not infected or clinically compressive [3,6,7,8]. Follow-up imaging confirmed that proximal diversion alone was sufficient for complete reabsorption of both collections.

### 3.5. Context Within the Published Literature

To contextualize the present case, a focused PubMed search updated on 28 May 2026 was performed using combinations of the terms “forniceal rupture”, “calyceal rupture”, “renal pelvis rupture”, “upper urinary tract rupture”, “urinary extravasation”, and “urinoma” with “urinary retention”, “bladder outlet obstruction”, “benign prostatic hyperplasia”, “postoperative urinary retention”, “neurogenic bladder”, and “high pressure chronic retention”. English-language case reports and case series were reviewed, and the reference lists of relevant articles were screened manually to identify additional retention- or outlet obstruction-related publications. The resulting reports most closely related to the present case are summarized in Table 2.

The published literature confirms that retention-related upper urinary tract rupture spans a spectrum rather than a single clinicoradiological entity. Urbain et al. described perirenal urinoma secondary to prostatic obstruction managed conservatively [12]. Choi et al. reported upper ureteric rupture in a woman with neurogenic urinary retention, managed with temporary percutaneous drainage, antibiotics, and subsequent stenting [13]. Sarmah et al. described proximal ureteric rupture from massive prostatic enlargement treated with catheter drainage and antibiotics [14]. Dakwar et al. reported unilateral renal forniceal rupture secondary to postoperative urinary retention [15]. Pang et al. described bilateral spontaneous urinary extravasation in a patient with benign prostatic hyperplasia, emphasizing that bilateral leakage may occasionally be self-limiting [16]. Maitra and Conn reported bilateral subcapsular urinomas in high-pressure chronic retention [17], and Deen et al. later described spontaneous ureteric rupture in high-pressure chronic retention managed with stenting and later outlet surgery [18].

Against this background, the present case appears exceptional for two reasons. First, the rupture was bilateral and anatomically forniceal rather than ureteric or merely radiographic extravasation. Second, the precipitating setting was acute urinary retention in an older man whose lower urinary tract was likely compromised by prior radiotherapy rather than by extreme benign gland enlargement. The PubMed search did not identify a prior report that clearly documented bilateral renal forniceal rupture directly attributable to acute urinary retention using this exact combination of clinical and imaging features. This claim should remain appropriately qualified by the search strategy used, but it supports the novelty of the present report and justifies its contribution to the literature.

### 3.6. Healing, Follow-Up, and Practical Lessons

The negative nephrostograms obtained immediately after PCN placement support the practical conclusion that urinary diversion reduced intraluminal pressure sufficiently for the leak to cease and become radiologically occult at the time of imaging. These findings may also reflect the well-established regenerative capacity of the urothelium. However, they do not establish the exact timing of healing and should therefore be interpreted with caution. Moreover, CT urography and nephrostography are not physiologically equivalent studies: contrast route, volume, timing, and pressure conditions differ substantially. The safer and more defensible interpretation is that upper tract diversion permitted spontaneous sealing of the ruptured fornices, after which no active leak was demonstrable under the conditions of nephrostogram.

Follow-up after the acute event is as important as the initial decompression. Once the leak resolves, the underlying cause of retention should be addressed so that recurrence does not occur. In the present case, normalization of renal function and successful removal of the nephrostomy tubes were only part of the therapeutic endpoint. Subsequent lower urinary tract assessment, including cystoscopy and residual-volume evaluation, was necessary to identify the likely outlet abnormality and to guide longer-term management. In similar cases, uroflowmetry, repeat post-void residual measurement, and formal urodynamics may be particularly useful when both structural obstruction and detrusor underactivity are plausible contributors.

Finally, the case highlights the need for careful inpatient monitoring after bladder decompression itself. Post-obstructive diuresis is common enough in admitted retention patients to demand active surveillance, and risk increases with large residual volumes and elevated creatinine [26,27]. Fluid replacement should be individualized, electrolytes followed serially, and renal function reassessed in tandem with symptom evolution [26,27]. This is especially important when the clinical course is complicated by urinary extravasation, because the apparent relief of obstruction at the bladder level does not necessarily mean that the upper tract injury has already resolved.

### 3.7. Limitations

As with many case reports, the mechanistic conclusions are inferential rather than experimentally proven. The exact pre-admission duration of elevated bladder pressure cannot be reconstructed with certainty, and the relative contributions of post-radiation outlet fibrosis and detrusor underactivity are not fully separable. Even with those limitations, the chronology, imaging, response to diversion, and exclusion of more common alternative causes provide a persuasive explanation for the event and yield practical lessons for clinicians managing retention with upper tract compromise.

## 4. Conclusions

This report describes an exceptionally rare case of spontaneous bilateral renal forniceal rupture associated with acute urinary retention in an elderly man with prior prostate radiotherapy. The report supports four practical messages. First, urinary retention accompanied by flank or abdominal pain, hydronephrosis, and acute kidney injury should raise suspicion for upper urinary tract rupture rather than being attributed reflexively to “simple” retention. Second, delayed-phase CT urography is critical for confirming rupture, defining its extent, and excluding competing causes such as stone disease or malignant obstruction. Third, in bilateral cases complicated by severe renal dysfunction, inflammatory response, or sizeable urinoma, active upper tract diversion with percutaneous nephrostomy is a rational and effective strategy. Fourth, a relatively small prostate in a previously irradiated patient should prompt consideration of post-radiation outlet pathology and/or detrusor dysfunction as the true substrate for retention. Early recognition, thoughtful imaging, and cause-directed follow-up are essential to preserve renal function and reduce the risk of recurrence.

## Figures and Tables

**Figure 1:**
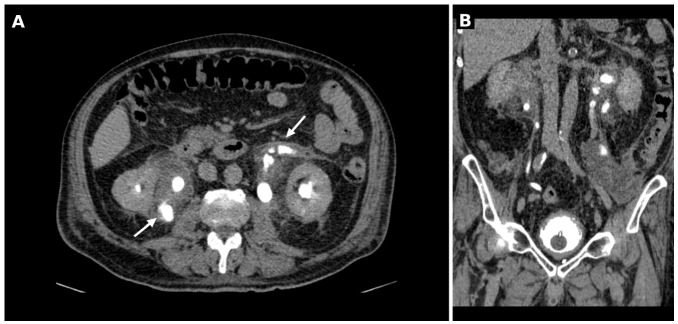
CT urography with excretory-phase imaging on hospital day 3. (**A**) Axial image showing severe bilateral hydronephrosis and bilateral contrast extravasation from the renal fornices. The left-sided leak is associated with a larger urinoma extending toward the left psoas compartment, whereas the right side shows a smaller perirenal collection. (**B**) Coronal pelvic image showing the Foley catheter within a decompressed/underdistended bladder, apparent circumferential bladder wall thickening/trabeculation, and no vesical contrast extravasation or bladder mass.

**Figure 2:**
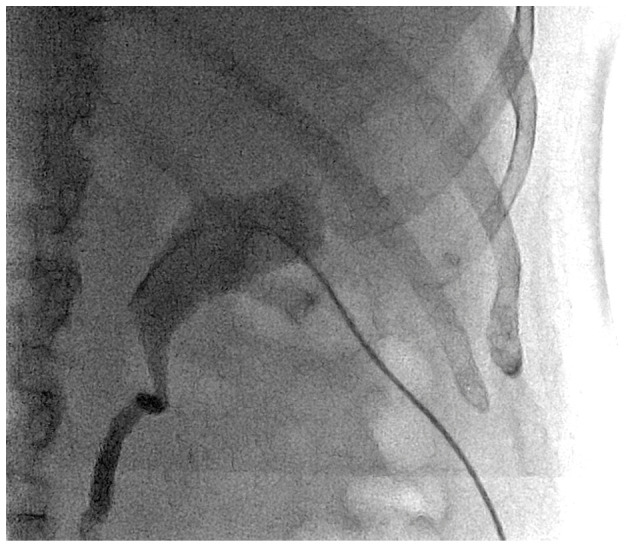
Right nephrostogram obtained immediately after placement of the left 8.5-Fr percutaneous nephrostomy tube on hospital day 4, showing opacification of the collecting system without active contrast extravasation.

**Figure 3:**
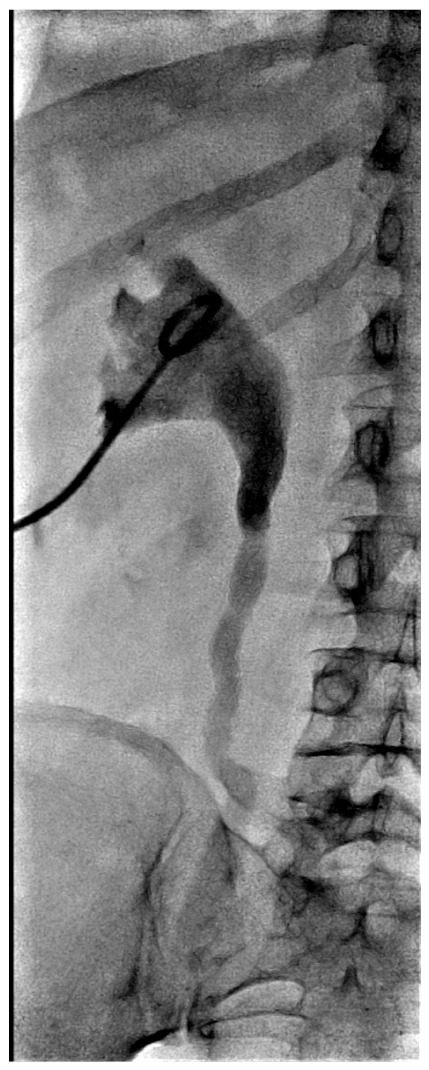
Left nephrostogram obtained immediately after placement of the right 8.5-Fr percutaneous nephrostomy tube on hospital day 4, again showing no active contrast extravasation.

**Table 1:** Timeline of presentation, investigation, treatment, and follow-up.

Hospital Day	Clinical Events/Investigations
Day 0	Emergency department presentation with lower abdominal and bilateral flank pain and inability to void; bedside ultrasound demonstrated urinary retention (~700 mL) and bilateral hydronephrosis. Foley catheter inserted; 900 mL drained. Admission creatinine 5.2 mg/dL; WBC 9.5 × 10^9^/L; CRP 117 mg/L.
Day 1	Post-obstructive diuresis after bladder decompression; strict urine output, fluid balance, and electrolyte monitoring. Peak urine output approximately 3.7 L/24 h. Creatinine improved to 3.4 mg/dL.
Day 2	Continued biochemical improvement with persistent bilateral flank pain despite bladder drainage. Creatinine improved to 2.1 mg/dL. Cultures remained negative.
Day 3	CT urography with delayed excretory phase demonstrated severe bilateral hydroureteronephrosis, bilateral renal forniceal rupture, and bilateral urinomas, including a larger left-sided collection extending toward the psoas. At the same CTU assessment, the Foley catheter was in situ in a decompressed bladder, and no vesical contrast extravasation was seen.
Day 4	Bilateral 8.5-Fr percutaneous nephrostomy tubes placed for upper tract diversion. Immediate bilateral nephrostography showed no active contrast extravasation; antegrade ureteral stents were not placed.
Day 7	Clinically stable and afebrile; inflammatory markers declining and renal function normalized. Discharged with Foley catheter and bilateral nephrostomy tubes in situ.
1 Month	Follow-up CT combined with nephrostography showed complete resolution of bilateral urinomas and no persistent leak; nephrostomy tubes removed. Follow-up creatinine remained normal.
6 Weeks	Outpatient cystoscopic reassessment suggested mild post-radiation outlet narrowing without fixed anterior urethral stricture; trial without catheter successful with low residual urine volume.

CRP, C-reactive protein; CT, computed tomography; WBC, white blood cell count.

**Table 2:** PubMed-indexed reports of retention- or bladder outlet obstruction-related upper urinary tract rupture, urinary extravasation, or urinoma relevant to the present case (search updated 24 March 2026).

Citation	Age/Sex	Retention/BOO Mechanism	Site and Laterality	Imaging	Treatment	Outcome
Urbain et al. [12]	NR/M	Prostatic obstruction/BPH	Caliceal fornix rupture with perirenal urinoma; laterality not clearly specified in abstract	Not clearly stated in abstract	Bladder decompression and conservative management	Resolution
Choi et al. [13]	75/F	Neurogenic bladder with urinary retention/detrusor underactivity	Right upper/proximal ureter; unilateral	CT plus antegrade pyelography	Foley catheter, 8.5-Fr PCN, antibiotics, then double-J stent	No leak after 7 days; resolved
Sarmah et al. [14]	77/M	Massive prostatic enlargement with chronic retention	Left proximal ureter; unilateral	Ultrasound plus delayed-phase CT	Urinary catheter and antibiotics; conservative management	Improved conservatively
Dakwar et al. [15]	43/F	Postoperative urinary retention	Right renal fornix; unilateral	CT plus pyelography/nephrogram	Bladder decompression and ureteral stenting via percutaneous access	Resolved
Pang et al. [16]	67/M	BPH with BOO/retention	Bilateral spontaneous urinary extravasation from collecting systems	CT urography plus pyelography	Conservative treatment and catheterization	Complete absorption at 14 days
Maitra and Conn [17]	69/M	High-pressure chronic retention due to BOO	Bilateral subcapsular urinomas	Ultrasound plus CT urogram	Catheterization; planned outlet surgery	Renal function normalized; follow-up incomplete
Deen et al. [18]	69/M	High-pressure chronic retention	Right ureter; unilateral	Non-contrast CT plus contrast study/follow-up imaging	Catheterization, JJ stent, later TURP	Improved
Present case	82/M	Acute urinary retention; likely post-radiation outlet dysfunction with possible detrusor underactivity	Bilateral renal fornices with bilateral urinomas	Ultrasound plus CT urography (excretory phase) plus nephrostography	Foley catheter and bilateral PCN	Complete radiological resolution at 1 month; PCNs removed

Abbreviations: BOO, bladder outlet obstruction; BPH, benign prostatic hyperplasia; CT, computed tomography; PCN, percutaneous nephrostomy; TURP, transurethral resection of the prostate; NR, not reported. Because bilateral outlet obstruction-related cases are exceptionally sparse, the table includes closely related reports of spontaneous urinary extravasation or urinoma when the underlying mechanism was urinary retention or bladder outlet obstruction.

## Data Availability

The data underlying this case report are not publicly available because they contain information that could compromise patient privacy. De-identified details may be available from the corresponding author on reasonable request and subject to institutional approval.

## Abbreviations

The following abbreviations are used in this manuscript:

AKI	Acute kidney injury
BOO	Bladder outlet obstruction
CT	Computed tomography
CTU	Computed tomography urography
EBRT	External beam radiotherapy
PCN	Percutaneous nephrostomy
POD	Post-obstructive diuresis
PVR	Post-void residual
